# All-treatment array of hepatocellular carcinoma from initial diagnosis to death: observation of cumulative treatments

**DOI:** 10.1007/s00432-017-2480-9

**Published:** 2017-07-25

**Authors:** Hae Moon, Ji Eun Choi, In Joon Lee, Tae Hyun Kim, Seong Hoon Kim, Young Hwan Ko, Hyun Boem Kim, Byung-Ho Nam, Joong-Won Park

**Affiliations:** 1 Department of Internal Medicine, National Cancer Center, Goyang, Republic of Korea; 2 Center for Liver Cancer, National Cancer Center, Goyang, Republic of Korea; 3 Department of Cancer Control and Policy, Graduate School of Cancer Science and Policy, National Cancer Center, Goyang, Republic of Korea

**Keywords:** Hepatocellular carcinoma, Cohort studies, Treatment outcome, Recurrence

## Abstract

**Purpose:**

In clinical practice, most patients with hepatocellular carcinoma require subsequent treatments for remaining, progressing, or recurring tumors. We investigated all-treatment array and outcomes in an HCC cohort from initial diagnosis to death.

**Methods:**

We enrolled 1687 consecutive patients with HCC who underwent initial diagnosis and treatment at the National Cancer Center, Korea, from January 2004 to December 2009.

**Results:**

In total, 1357 patients (80.4%) showed RPRTs during median 20.4-month follow-up. Initial transplantation resulted in the least rate (32.3%) of RPRTs. Median treatment frequency was 3.0 times (range 1–20) and 382 patients (27.3%) received treatments ≥6 times. The median treatment frequency was different based on four factors (*p* < 0.05): age, tumor stage, tumor type and initial treatment modality. Patients with Barcelona Clinic Liver Cancer stage 0 received less frequent treatments. As the stage progressed from 0 to B, the median treatment frequency increased. Radiofrequency ablation as initial treatment was associated with the longest median treatment interval at 19.0 weeks, followed by resection at 14.1 weeks. The median treatment interval was significantly shorter as the stage progressed (*p* < 0.01). TACE was most frequently performed for RPRTs; the median number of subsequent TACE was 3 (range 1–19). Subsequent treatment array was very heterogeneous, and no certain pattern was found.

**Conclusions:**

Our findings suggest that the survival outcome of patients with HCC is based on the results of cumulative multiple treatments rather than an initial treatment. It is time to consider prospective studies evaluating sequential treatment array of HCC.

## Introduction

Hepatocellular carcinoma (HCC) is the sixth most frequent tumor worldwide, and the second most common cause of cancer-related death (Ferlay et al. 2015). Surgical resection, liver transplantation, and loco-regional treatments including radiofrequency ablation (RFA) are recommended as curative treatments for HCC (Korean Liver Cancer Study Group (KLCSG), National Cancer Center, Korea (NCC) 2015; Bruix and Sherman 2011; European Association for the Study of the Liver, European Organisation for Research and Treatment of Cancer 2012). However, only one-third of HCC patients are possible candidates for these curative treatments (European Association for the Study of the Liver, European Organisation for Research and Treatment of Cancer 2012) and the remaining 60–70% of patients receive non-curative treatments such as transarterial chemoembolization (TACE) or sorafenib as initial therapy (Bruix and Sherman 2011; European Association for the Study of the Liver, European Organisation for Research and Treatment of Cancer 2012). Globally, TACE is the most frequently used initial treatment for unresectable HCC (Ikai et al. 2005; Park et al. 2015; Takayasu et al. 2006). Unfortunately, the outcome for patients with HCC treated with either curative or palliative modalities is not satisfactory. Such poor prognosis of HCC is partly due to tumor characteristics, which are referred to as frequent remaining, progressing, or recurring tumors (RPRT) after treatment (Kim et al. 2014).

All guidelines and most treatment outcome studies have been established based only on the initial treatment modality (Korean Liver Cancer Study Group (KLCSG), National Cancer Center, Korea (NCC) 2015; Bruix and Sherman 2011; European Association for the Study of the Liver, European Organisation for Research and Treatment of Cancer 2012; Kudo et al. 2011). However, HCC frequently recurs with a 5-year recurrence rate of 70% even after curative liver resection (Llovet 2005). Therefore, in clinical practice most patients with HCC require subsequent treatments for RPRT. Appropriate subsequent treatment is just as important as initial treatment for improving patient survival in HCC. Unfortunately, there are no prospective studies on the best sequencing of treatment options for RPRT and even no longitudinal cohort study based on overall real-practice scenarios in patients with HCC. We, for the first time, investigated the patterns of all treatments provided to patients and outcomes of RPRTs in an HCC cohort from initial diagnosis to death at a single referral institution in South Korea.

By evaluating the results of cumulative treatments in the real-world management, healthcare providers can recognize the limited effects of single treatment and the meaning of initial treatment-based survival outcome in patients with HCC, and could improve the design of clinical trials and therapeutic strategies.

## Materials and methods

### Data source

**Fig. 1:**
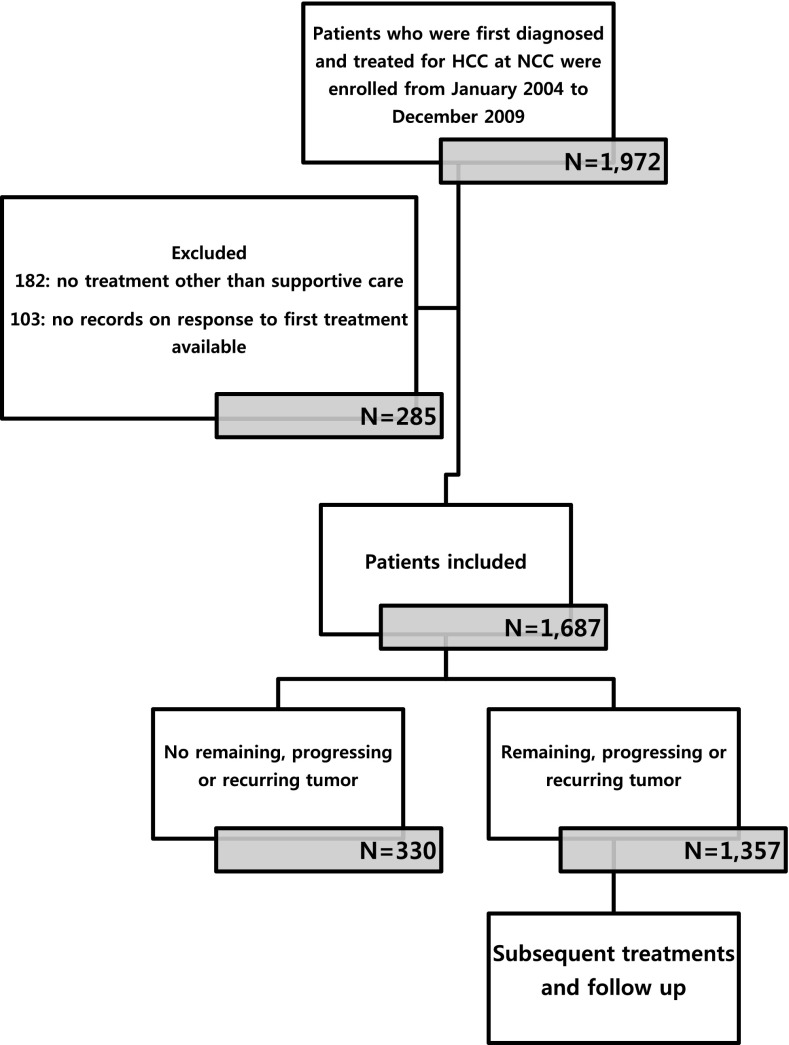
Flow chart of the study scheme. *HCC* hepatocellular carcinoma, *NCC* National Cancer Center, Korea

The National Cancer Center, Korea launched a cohort as of January 2004 in an attempt to carry out research on patients receiving an initial diagnosis and treatment of HCC at the institution (Kwak et al. 2014). The initial treatment was applied to most patients according to Korean guidelines (Korean Liver Cancer Study Group (KLCSG), National Cancer Center, Korea (NCC) 2015). Imposing a cut-off of December 31, 2009, 1972 patients were identified prospectively and their relevant data were extracted retrospectively from medical records (Fig. 1).

Of these patients, 285 were not enrolled based on the exclusion criteria: (1) no treatment other than best supportive care or palliative pain control radiotherapy to metastases outside the liver (182 patients) and (2) inability to evaluate tumor response to first treatment (103 patients). As a result, the study enrolled 1687 patients, 1357 of whom experienced RPRT in the follow-up period. On each confirmed RPRT occurrence, the interdisciplinary team provided patients with the next line treatment as appropriate, taking into account a range of factors, including the number and size of lesions, invasion to major vascular or biliary structures, and whether disease was confined to the liver or metastatic, residual liver function, and comorbidities. Information with respect to treatment was collected beginning with the diagnosis of HCC, including sequence of treatments, modality of treatment, and dates of treatment initiation and completion in case of cytotoxic chemotherapy or sorafenib (hereafter systemic chemotherapy). In case of combined-modality treatments, e.g., TACE + radiotherapy, each was counted individually.

Clinical outcomes were also analyzed, including date of death and dates of any clinically diagnosed RPRT based on imaging studies, including change in lesion size and radiocontrast media enhancement, appearance of new lesions, or symptomatology. Patients were grouped on the basis of their initial treatment. The array of treatments was sequenced for every single patient who developed RPRT and the all-sequenced data were analyzed to gain detailed insight into the nature of treatment complexity. The institutional review board of the National Cancer Center, Korea approved this study (NCC2016-029), which complied with the International Ethical Guidelines for Biomedical Research Involving Human Subjects, Good Clinical Practice Guidelines, the Declaration of Helsinki, and local rules and regulations.

### Statistical analysis

Descriptive statistics were used to present demographic and clinical characteristics. To compare groups, the one-way ANOVA test, Mann–Whitney *U* test, or Kruskal–Wallis test were used as appropriate. Kaplan–Meier curves for time to an event were constructed and compared with the use of the log-rank test regarding overall survival. All data were analyzed using STATA. A *p* value less than 0.05 was considered statistically significant in all analyses.

## Results

### Patient characteristics

For the enrolled 1687 patients, follow-up visits no later than December 31, 2012 were considered the end of observation. Median follow-up duration was 20.4 months (Fig. 1). Table 1 shows baseline demographic and clinical characteristics of the study population. Median patient age was 56 years (interquartile range 46–66 years), with 82.8% being male. Concerning risk factors, hepatitis B virus (HBV) accounted for 1249 (74.0%) cases of HCC. According to the Barcelona Clinic of Liver Cancer (BCLC) stage, most patients were diagnosed as stage C (61.2%), followed by stage A (22.6%); with respect to the modified UICC stage, 33.4% patients were stage II and 30.6% patients were stage IV.

**Table 1:** Baseline characteristics of the patients (*n* = 1687)

Characteristics	No. of patients (%)
Median age (interquartile range) (years)	56 (46–66)
Etiology	
HBV	1249 (74.0)
HCV	164 (9.7)
Alcohol	134 (7.9)
Cryptogenic	140 (8.3)
Child–Pugh class	
A	1476 (87.5)
B	207 (12.3)
C	4 (0.2)
BCLC stage	
0	92 (5.5)
A	381 (22.6)
B	173 (10.3)
C	1032 (61.2)
D	9 (0.5)
mUICC stage	
I	170 (10.1)
II	564 (33.4)
III	437 (25.9)
IVa	332 (19.7)
IVb	184 (10.9)
Tumor type	
Well-defined	1216 (72.1)
Ill-defined	471 (27.9)

*HBV* hepatitis B virus, *HCV* hepatitis C virus, *BCLC* Barcelona Clinic Liver Cancer, *mUICC* modified Union of International Cancer Control

### Frequency and interval of treatment

Out of 1687 patients, 1357 patients (80.4%) showed PRRTs during the observation period (Fig. 1). Regarding the initial treatment, TACE was performed for 1089 patients (64.6%), followed by surgical resection (*n* = 367, 21.8%), systemic chemotherapy (*n* = 77, 4.6%), radiotherapy (*n* = 71, 4.2%), RFA/percutaneous ethanol injection (PEI) (*n* = 52, 3.1%), and transplantation (*n* = 31, 1.8%). In patients who showed RPRTs, median treatment frequency (mTF) was 3.0 times (range 1–20) and 382 patients (27.3%) received six or more treatments; a six of treatment frequency correspond three-quantile. The mTF was statistically different based on four factors (*p* < 0.05): age, tumor stage (BCLC, and mUICC), and tumor type, and initial treatment modality (*p* < 0.01) (Table 2). Patients with ill-defined type HCC survived shorter and less frequent treatment. Patients aged above 60 years, BCLC stage B, mUICC stage II or III, or with well-defined HCC had a greater number of subsequent treatments; patients with BCLC stage 0 or mUICC stage I received less frequent treatments.

**Table 2:** Survival time and frequency of treatment according to baseline characteristics in patients with RPRTs (*n* = 1357)

Baseline characteristics	Median survival, months (95% CI)	Median frequency of treatment (range)	*p* value*
Age (years)			<0.01
<40	12.3 (6.7–17.8)	3.0 (1–16)	
40–60	20.6 (18.1–23.1)	3.0 (1–18)	
≥60	26.9 (24.7–29.1)	4.0 (1–20)	
Sex			0.52
Male	22.0 (20.0–23.9)	3.0 (1–20)	
Female	29.9 (23.5–36.2)	3.0 (1–16)	
Etiology			0.41
Viral	22.5 (20.5–24.6)	3.0 (1–20)	
Non-viral	27.7 (23.9–31.4)	4.0 (1–17)	
Child–Pugh class^a^			0.09
A	24.0 (21.8–26.2)	3.0 (1–20)	
B	18.9 (15.3–22.4)	3.0 (1–16)	
C	6.2 (4.1–8.2)	2.0(1–7)	
BCLC			<0.01
0	92.1 (43.3–141.0)	3.0 (1–12)	
A	52.5 (48.1–56.9)	4.0 (1–15)	
B	32.1 (27.6–36.5)	5 (1–20)	
C	14.8 (13.1–16.5)	3 (1–20)	
mUICC			<0.01
I	67.1 (43.3–91.0)	3.0 (1–17)	
II	42.4 (37.4–47.4)	4.0 (1–16)	
III	24.4 (21.8–27.0)	4.0 (1–20)	
IV	8.7 (7.8–9.6)	3.0 (1–20)	
Tumor type			<0.01
Well-defined	33.9 (30.5–37.3)	4.0 (1–20)	
Ill-defined	8.4 (7.5–9.3)	2.0 (1–14)	
Initial modality			<0.01
Resection	52.5 (41.8–63.2)	4 (1–16)	
RFA/PEI	50.2 (30.8–69.6)	3 (1–14)	
TACE	22.5 (20.3–24.7)	4 (1–20)	
EBRT	10.0 (7.4–12.6)	2 (1–14)	
Systemic chemotherapy	5.8 (4.6–7.0)	1 (1–11)	

*CI* confidential interval, *HBV* hepatitis B virus, *HCV* hepatitis C virus, *BCLC* Barcelona Clinic Liver Cancer, *mUICC* modified Union of International Cancer Control, *RFA* radiation frequency ablation, *PEI* percutaneous ethanol injection, *TACE* transarterial chemoembolization, *EBRT* external beam radiation therapy

* *p* value for treatment frequency

^a^ Child C was excluded from statistical tests because the number of patients was four

As the BCLC stage progressed from 0 to B, the median survival time was shortened and mTF increased; however, stage C had a shorter survival time and fewer treatments than other stages. Patients who underwent resection as an initial treatment showed longer survival, but had mTF four times. In addition, patients with well-preserved liver function as Child–Pugh class A tended to receive more treatments in comparison to patients with poor liver function of Child–Pugh class B and C, although this was not statistically significant (*p* = 0.09). We evaluated the interval between treatments according to the initial modality and tumor stage (Table 3).

**Table 3:** Treatment interval according to the initial treatment modalities and stages in patients who showed RPRTs (*n* = 1357)

	Median interval between treatments (range Q1, Q3) (weeks)	*p* value
Initial modality		<0.01
Resection	14.1 (5.9, 32.7)	
RFA/PEI	19.0 (7.9, 40.1)	
TACE	9.3 (5.1, 22.1)	
EBRT	7.9 (5.0, 16.0)	
Systemic chemotherapy	7.9 (2.5, 22.5)	
BCLC stage		<0.01
0	21.7 (9.4, 52.3)	
A	16.7 (6.4, 36.4)	
B	10.3 (5.1, 22.1)	
C	8.0 (4.9, 19.9)	
mUICC		<0.01
I	20.4 (8.0, 42.1)	
II	13.9 (6.0, 32.7)	
III	9.1 (5.1, 21.9)	
IV	6.6 (4.6, 11.9)	

*RPRTs* recurring, progressing, or remaining tumors, *RFA* radiation frequency ablation, *PEI* percutaneous ethanol injection, *TACE* transarterial chemoembolization, *EBRT* external beam radiation therapy, *BCLC* Barcelona Clinic Liver Cancer, *mUICC* modified Union of International Cancer Control

Initial treatment with RFA was associated with the longest median treatment interval (mTI) at 19.0 weeks, followed by resection at 14.1 weeks. With both BCLC and mUICC stages, the mTI was significantly shorter as the stage progressed (*p* < 0.01).

### All-treatment array

**Fig. 2:**
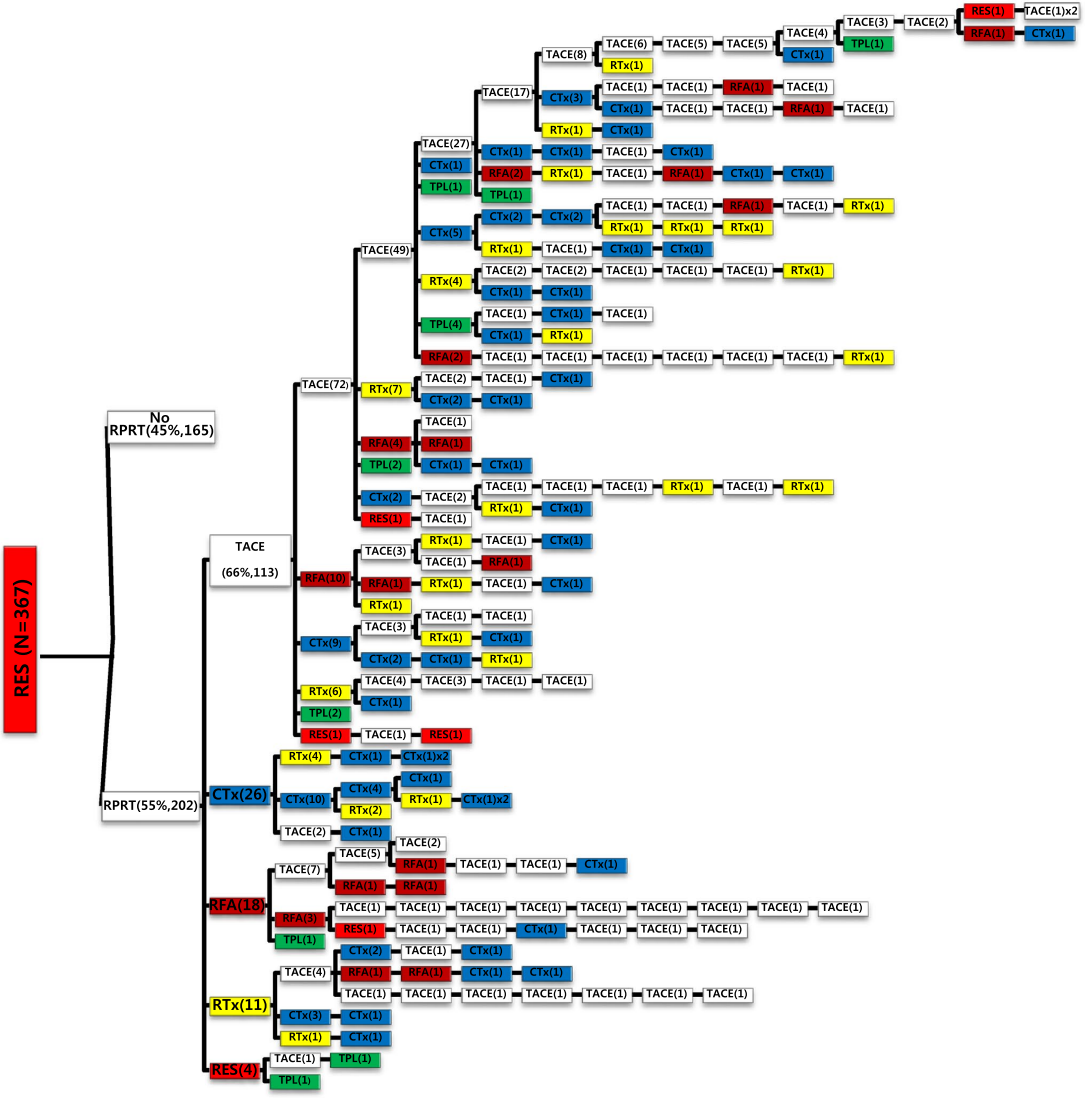
Tree diagram showing treatment sequences following resection as the initial treatment. Figures in *parentheses* indicate the number of cases. Each *color* (*white*, *blue*, *green*, *yellow*, *red*, and *brown*) represents a specific treatment modality. RES (*red*), resection; RFA (*brown*), radiofrequency ablation or percutaneous ethanol injection; RTx (*yellow*), EBRT; CTx (*blue*), systemic chemotherapy including sorafenib; TPL (*green*), transplantation; TACE (*white*), transarterial chemoembolization; RPRT, remaining, progressing, or recurrent tumor

We examined all-treatment array according to each treatment modality as the initial treatment. In case of surgical resection as the initial treatment (Fig. 2), 202 out of 367 patients (55%) experienced RPRT during the observation period and required a second treatment, the highest proportion of which was TACE (66%) followed by systemic chemotherapy (15%), local ablation (11%), external beam radiation therapy (EBRT) (6.4%), and resection (2.3%). One hundred thirty-seven out of 367 patients (37.3%) had a third treatment after initial surgical resection, and TACE was performed most often in 86 patients (62.8%). The median number of subsequent TACE sessions after resection was 3 (range 1–14) (Table 4).

**Table 4:** The number of patients according to frequency of TACE sessions after each first treatment modality in patients with RPRTs (*n* = 1357)

Initial treatment	TACE session frequency																				
	None	1	2	3	4	5	6	7	8	9	10	11	12	13	14	15	16	17	18	19	Median no. (range)
Resection (*n* = 202)	73	37	25	26	11	14	3	4	2	3	1	1	1	0	1	0	0	0	0	0	3 (1–14)
RFA/PEI (*n* = 31)	10	10	3	3	2	1	1	0	0	1	0	0	0	0	0	0	0	0	0	0	2 (1–9)
TACE (*n* = 977)	283	181	136	101	69	53	50	27	24	16	14	9	6	3	4	0	0	0	0	1	3 (1–19)
EBRT (*n* = 65)	36	12	5	6	2	2	0	0	2	0	0	0	0	0	0	0	0	0	0	0	2 (1–8)
Chemotherapy (*n* = 72)	58	6	3	2	1	0	1	0	0	0	1	0	0	0	0	0	0	0	0	0	2 (1–10)
Total (*n* = 1357)	464	250	173	138	85	70	55	31	29	20	16	10	7	3	5	0	0	0	0	1	3 (1–19)

*RPRTs* recurring, progressing, or remaining tumors, *RFA* radiation frequency ablation, *PEI* percutaneous ethanol injection, *TACE* transarterial chemoembolization, *EBRT* external beam radiation therapy

**Fig. 3:**
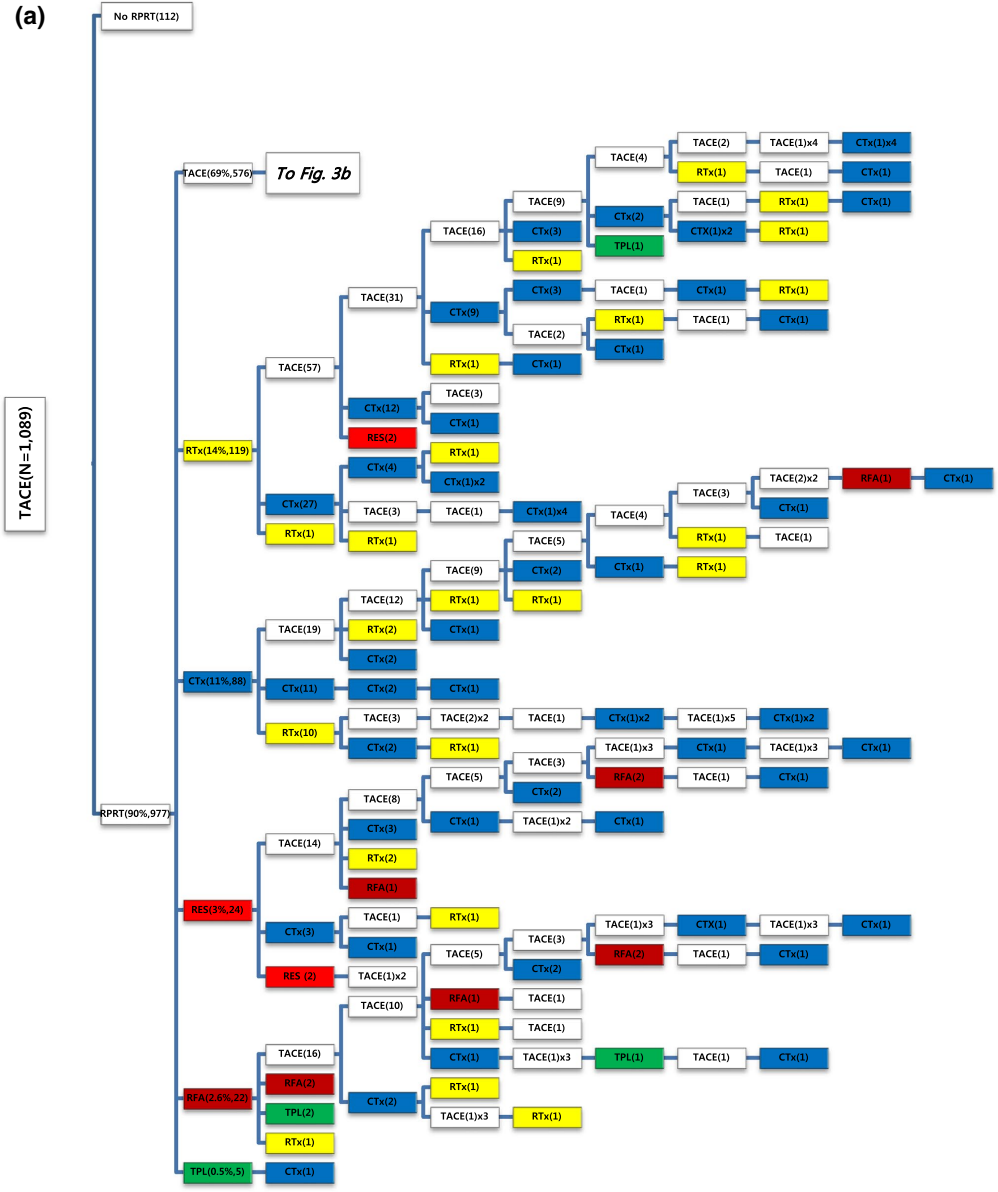

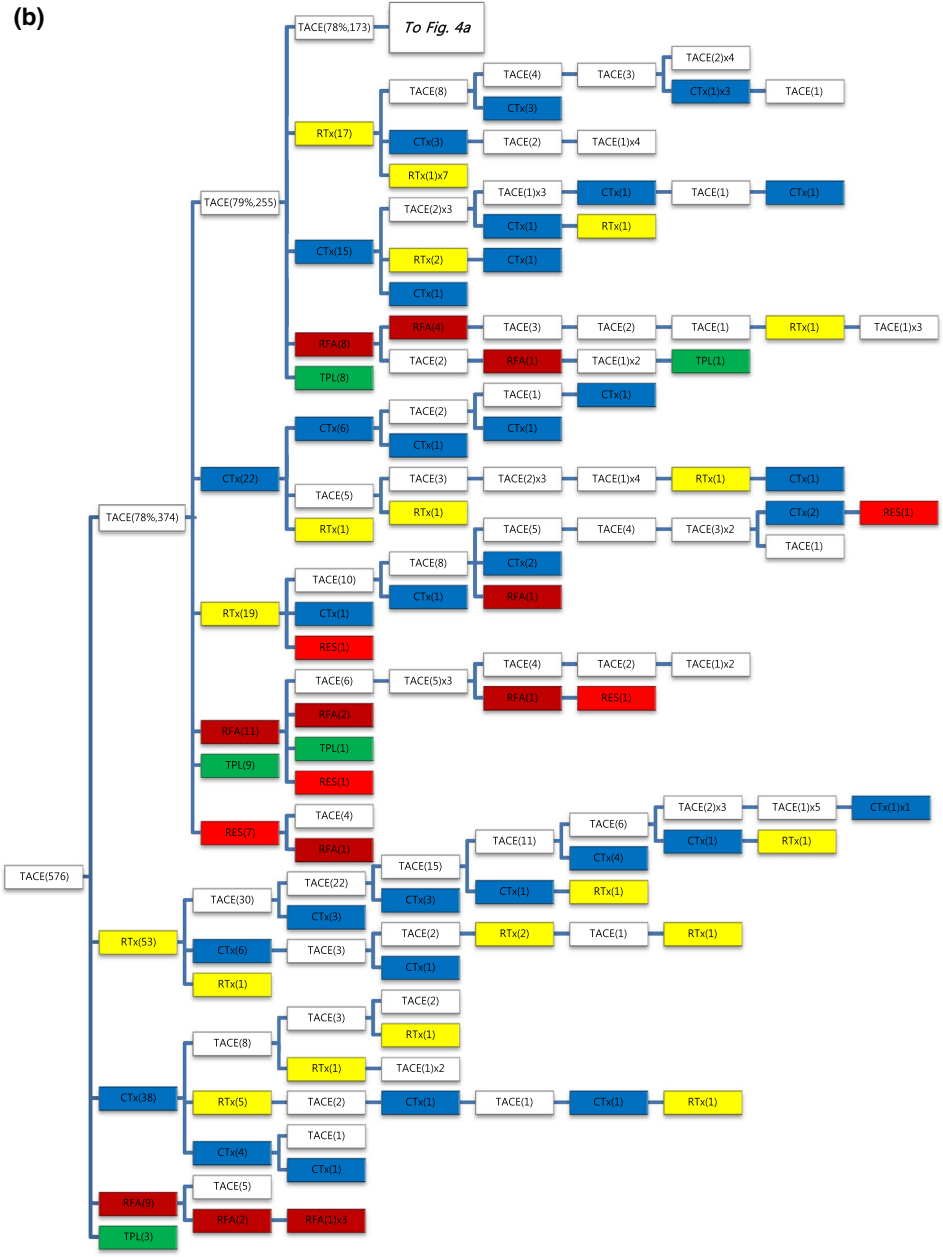
**a**, **b** Tree diagram showing treatment sequences following TACE as the initial treatment. Figures in *parentheses* indicate the number of cases. Treatment array is too long to be expressed in one figure; it is expressed as separate figures **a**, **b**. Each *color* (*white*, *blue*, *green*, *yellow*, *red*, and *brown*) represents a specific treatment modality. RES (*red*), resection; RFA (*brown*), radiofrequency ablation or percutaneous ethanol injection; RTx (*yellow*), EBRT; CTx (*blue*), systemic chemotherapy including sorafenib; TPL (*green*), transplantation; TACE (*white*), transarterial chemoembolization; RPRT, remaining, progressing, or recurrent tumor

**Fig. 4:**
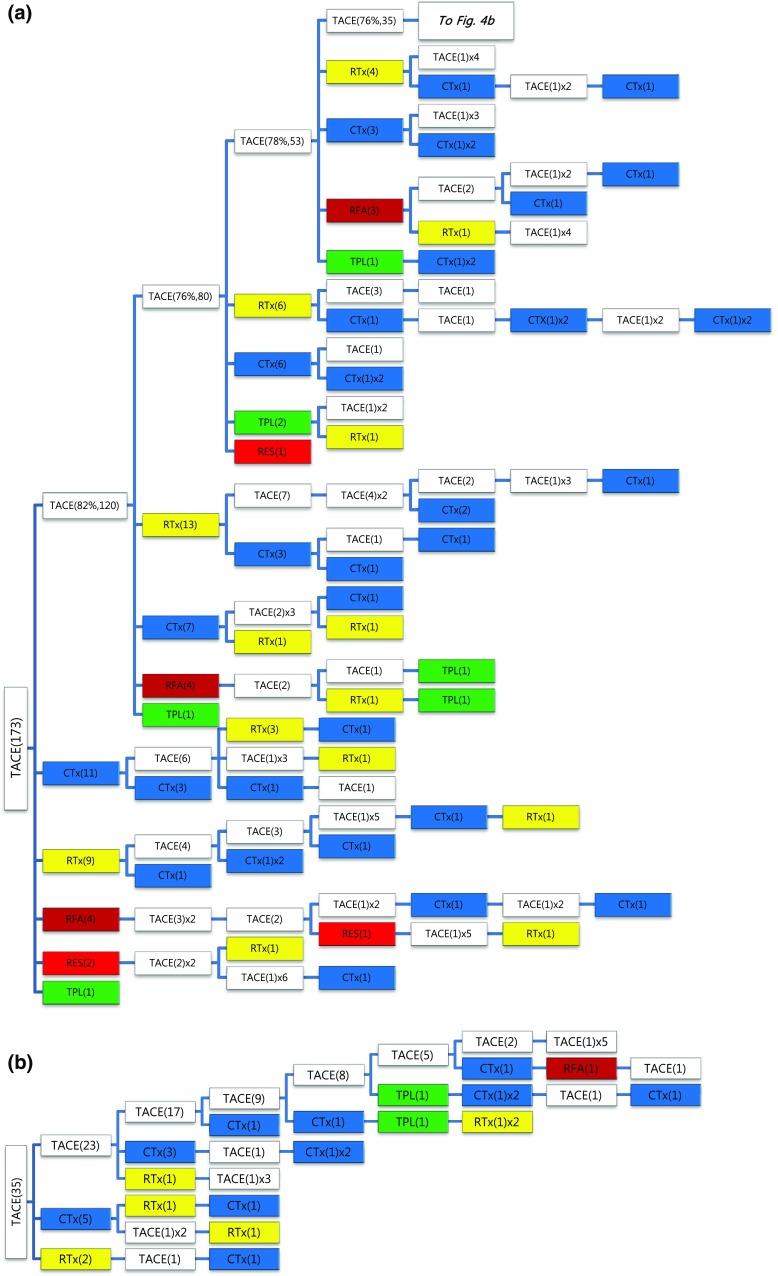
**a**, **b** Tree diagram showing treatment sequences following TACE as the initial treatment (continued from Fig. 3b). Figures in *parentheses* indicate the number of cases. Treatment array is too long to be expressed in one figure; it is expressed as separate figures **a**, **b**. Each *color* (*white*, *blue*, *green*, *yellow*, *red*, and *brown*) represents a specific treatment modality. RES (*red*), resection; RFA (*brown*), radiofrequency ablation or percutaneous ethanol injection; RTx (*yellow*), EBRT; CTx (*blue*), systemic chemotherapy including sorafenib; TPL (*green*), transplantation; TACE (*white*), transarterial chemoembolization; RPRT, remaining, progressing, or recurrent tumor

Of the 1089 patients who received TACE as initial treatment, 977 patients (90%) needed a second treatment for RPRT (Fig. 3a). Treatment array after initial TACE is demonstrated in Figs. 3a, b and 4a, b. As the second treatment after initial TACE, TACE, EBRT, systemic chemotherapy, surgical resection, and local ablation were performed in 69, 14, 11, 3, and 2.6% of patients, respectively. The majority of patients who experienced RPRT after a second treatment required a third treatment; TACE, EBRT, systemic chemotherapy including sorafenib, local ablation, and liver transplantation was performed in 78, 11, 8, 2, and 1%, respectively, as the third treatment (Fig. 3a, b). The median number of subsequent TACE sessions after initial TACE was 3 (range 1–19). One patient with BCLC stage C, treated initially with TACE, was managed with 19 additional TACE sessions in succession while surviving 71.9 months (Fig. 4a, b; Table 4).

**Fig. 5:**
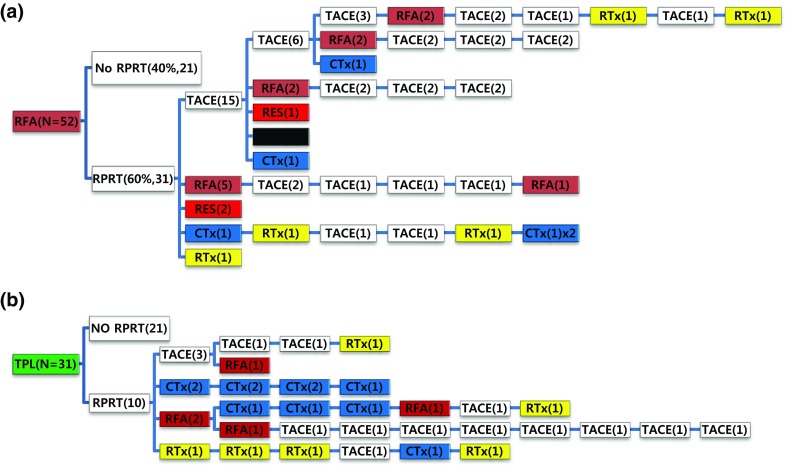
Tree diagram showing treatment sequences following **a** RFA/PEIT, **b** transplantation. Figures in *parentheses* indicate the number of cases. Each *color* (*white*, *blue*, *green*, *yellow*, *red*, and *brown*) represents a specific treatment modality. RES (*red*), resection; RFA (*brown*), radiofrequency ablation or percutaneous ethanol injection; RTx (*yellow*), EBRT; CTx (*blue*), systemic chemotherapy including sorafenib; TPL (*green*), transplantation; TACE (*white*), transarterial chemoembolization; RPRT, remaining, progressing, or recurrent tumor

Out of 52 patients managed with initial local ablation (RFA/PEI), 31 patients (60%) experienced RPRT and 24 of them received a second treatment (Fig. 5a). TACE was the most frequent intervention (62.5%) as the second treatment to control RPRT, followed by local ablation (20.8%), surgical resection (8.3%), systemic chemotherapy (4.2%), and EBRT (4.2%). Thirty-one patients received liver transplantation as the initial treatment (Fig. 5b). Ten patients (32.3%) developed RPRT during the observation period; three were treated with TACE, two with systemic chemotherapy, two with local ablation, and one with EBRT as the second treatment.

**Fig. 6:**
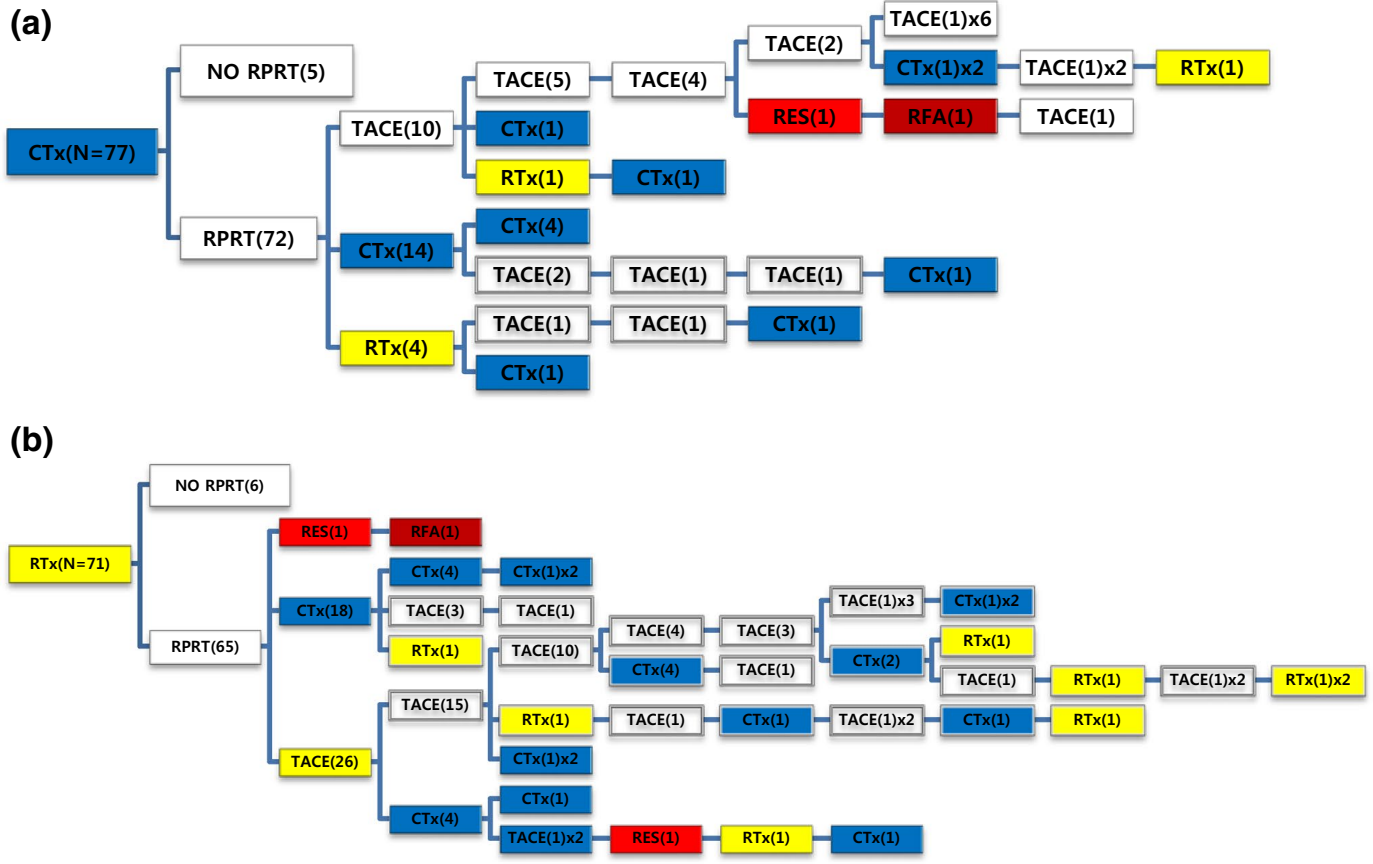
Tree diagram showing treatment sequences following **a** systemic chemotherapy or **b** EBRT as the initial treatment. Figures in *parentheses* indicate the number of cases. Each *color* (*white*, *blue*, *green*, *yellow*, *red*, and *brown*) represents a specific treatment modality. RES (*red*), resection; RFA (*brown*), radiofrequency ablation or percutaneous ethanol injection; RTx (*yellow*), EBRT; CTx (*blue*), systemic chemotherapy including sorafenib; TPL (*green*), transplantation; TACE (*white*), transarterial chemoembolization; RPRT, remaining, progressing, or recurrent tumor

Out of 77 patients managed with systemic chemotherapy as the initial treatment, 72 patients (93.5%) experienced RPRT; 28 patients (38.9%) received a second treatment and 15 patients (20.8%) received a third treatment (Fig. 6a). One patient received an additional 10 sessions of TACE after initial systemic chemotherapy (Table 4). Seventy-one patients received EBRT as the initial treatment (Fig. 6b). Sixty-five patients (91.5%) showed RPRTs; 45 patients (63.4%) had a second treatment and 28 patients (39.4%) had a third treatment.

TACE was the most commonly used therapy for RPRT after all initial treatment methods. Analysis of the frequency of TACE after the initial treatment showed that the mTF with TACE was 3 (range 1–19) (Table 4). Even in patients who received systemic chemotherapy as initial treatment, 14 patients (19.4%) had a median of 2 TACE sessions (range 1–10).

## Discussion

This study clearly demonstrates that most patients with HCC receive multiple treatments from diagnosis to death and the treatment array is very heterogeneous. After the first report of a second treatment in a global observation study (Park et al. 2015), information on the treatment patterns in patients with HCC remained poorly understood. In this cohort, 80.4% of patients received second or further treatments to control RPRTs; the mTF was 3.0 (range 1–20) during a median follow-up of 20.4 months (Table 2). This cohort study was performed at a referral center (Kwak et al. 2014), and so, there were a large number of patients with BCLC stage C, who had less frequent treatments and shorter survival time than patients with BCLC stage 0–B. The patients who received more treatment sessions showed a tendency toward longer survival (data not shown); however, this study was observational and so the frequency of treatments seems to only suggest that the patients had a medical condition that could be treated. However, patients with BCLC stage 0 had the longest survival period, but the mTF was the smallest and mTI was the longest (Tables 1, 2, 3). As stage progressed, the survival period decreased and the treatment frequency increased; patients with BCLC stage B had the highest number of treatments. However, patients with stage C had the shortest survival period and fewer treatments. This suggests that as the stage progresses, tumor progression may be accelerated due to changes in tumor biology; additional treatments may have limited effect due to deterioration of liver function and/or performance status after treatment and to the absence of appropriately effective modalities. Cheng et al. assumed that small HCCs may have either Gompertzian type of growth, in which the initial exponential growth decreases as tumor size increases, or rapid exponential growth (Cheng et al. 2002). This study seems to support a rapid exponential growth. Both tumoral and extratumoral factors determine the growth rate and biological aggressiveness of HCC (Trevisani et al. 2008). The ill-defined type HCC, recognized to have a poor prognosis (Demirjian et al. 2011), was associated with the shortest survival time and the fewest number of treatments; in multivariate analysis, tumor type appeared to be the most potent, but no statistical significance was found (Table 2).

Analysis of the treatment interval also supports the assumption concerning accelerated tumor progression. That is, as stage progresses, the treatment interval becomes significantly shorter (Table 3). As the BCLC stage progressed from 0 to C, the mTI decreased from 21.7 to 8.0 weeks. Because this cohort was collected from a single institution and treatment followed the Korean guidelines (Korean Liver Cancer Study Group (KLCSG), National Cancer Center, Korea (NCC) 2015), most patients were followed-up relatively regularly; the follow-up period was 1–2 months after the initial treatment, 2–4 months without RPRT, and 6 months after complete remission for 2 years. The difference in the treatment interval associated with the initial treatment method seems to be due to differences in disease stage at initial treatment (Table 3).

Subsequent treatment array was very heterogeneous, and this observational study could not suggest a certain pattern of subsequent treatment; a descriptive array was observed (Figs. 2, 3, 4, 5, 6). Widely heterogeneous RPRTs and the lack of evidence-based guidelines for subsequent treatment are causes for these various treatments arrays. In general, second and subsequent treatments are suggested to be based on initial treatment guidelines (Bruix and Sherman 2011; European Association for the Study of the Liver, European Organisation for Research and Treatment of Cancer 2012), but this lacks solid evidence. Prospective controlled studies are needed to guide subsequent treatment. Recently, a prognostic prediction model for second treatment outcome has been proposed (Choi et al. 2016) and will be useful for further study.

Similar to results from a global observational study (Park et al. 2015), TACE was the most common initial treatment (*n* = 1089, 64.6%) in our population, followed by surgical resection (*n* = 367, 21.8%). TACE is also the most commonly used second therapy for RPRTs after initial treatment of all methods; the proportion of TACE as second treatment was 66, 69, and 62.5% after initial resection, TACE, and RFA/PEI, respectively. The mTF with TACE was 3 (range 1–19) (Table 4). In this cohort, all TACE was performed on demand. Repeated administration of TACE may mean TACE failure or refractoriness, and other alternative treatments such as sorafenib may be necessary. Recently, there have been studies and reviews of TACE failure/refractoriness (Cheng et al. 2014; Kim et al. 2012; Kudo et al. 2014b; Yamanaka et al. 2012), but there is still no global consensus on the definition of TACE failure/refractoriness. In the Western world, a second TACE treatment is not recommended if there is a decrease in hepatic function after the first TACE (Hucke et al. 2014). However, in Asia including Korea or Japan, where TACE is performed with a superselection technique, significant deterioration of hepatic function after TACE is rare (Chung et al. 2011; Kudo et al. 2014a; Takayasu et al. 2006). Therefore, TACE could be applied repeatedly for the treatment of RPRTs if there is no stage progression. Some patients may be treated with TACE even if stage progression occurs (Chung et al. 2011).

In this cohort, RFA cases were relatively rare because National Insurance reimbursement was not feasible at that time. Recently, the number of cases of RFA as the initial treatment has been rapidly increasing after an insurance policy change. Transplantation as an initial treatment is also relatively rare in this cohort. In Korea, liver transplantation may not be acceptable to patients as an initial treatment because most cases are living-donor transplantation, which is provided by the family (Chen et al. 2013; Lee et al. 2016). After systemic chemotherapy including sorafenib, most patients have experienced RPRT and most have several subsequent treatments with other cytotoxic chemotherapy agents or TACE; about 40 and 20% of patients received a second and a third treatment, respectively (Fig. 6a; Table 4). In the recent cohort under construction, changes in the era of sorafenib are noted concerning systemic chemotherapy. Now that regorafenib has succeeded as a second-line treatment after sorafenib (Bruix et al. 2017), a major change in the subsequent treatment pattern is expected.

Although this study has certain limitations in that it is a practice-based observational study with wide heterogeneity of RPRTs, our findings provide the novel insight that the outcome of HCC is a result of cumulative multiple treatments. Our results provide a key cornerstone to discuss what the primary goal of treatment in HCC should be. The Panel of Experts in HCC-Design Clinical Trials recommend that the primary endpoint for phase 3 studies that assess primary HCC treatment is survival and clinical trials of locoregional therapies should report a time-to-local recurrence endpoint (Llovet et al. 2008). As seen in this study, time-to-local recurrence, or recurrence-free survival rather than overall survival seems to be more meaningful in the efficacy evaluation of local treatment, if we recognize that in real-world clinical practice, multiple subsequent treatments are provided after the initial local treatment. Even in patients managed with initial systemic chemotherapy, a part of patients received multiple subsequent treatments. It is time to actively consider prospective studies evaluating sequential and/or combination treatment of HCC.

## References

[CR1] Korean Liver Cancer Study Group (KLCSG), National Cancer Center, Korea (NCC) (2015). 2014 KLCSG-NCC Korea practice guideline for the management of hepatocellular carcinoma. Gut liver.

[CR2] Bruix J, Sherman M (2011). Management of hepatocellular carcinoma: an update Hepatology.

[CR3] Bruix J (2017). Regorafenib for patients with hepatocellular carcinoma who progressed on sorafenib treatment (RESORCE): a randomised, double-blind, placebo-controlled, phase 3 trial. Lancet.

[CR4] Chen CL, Kabiling CS, Concejero AM (2013). Why does living donor liver transplantation flourish in Asia?. Nat Rev Gastroenterol Hepatol.

[CR5] Cheng SJ, Freeman RB, Wong JB (2002). Predicting the probability of progression-free survival in patients with small hepatocellular carcinoma. Liver Transplant Off Publ Am Assoc Study Liver Dis Int Liver Transplant Soc.

[CR6] Cheng AL (2014). Re-evaluating transarterial chemoembolization for the treatment of hepatocellular carcinoma: consensus recommendations and review by an International Expert Panel. Liver Int.

[CR7] Choi SI, Yu A, Kim BH, Ko EJ, Park SS, Nam BH, Park JW (2016). A model predicting survival of patients with recurrent or progressive hepatocellular carcinoma: the MORE score. J Gastroenterol Hepatol.

[CR8] Chung GE (2011). Transarterial chemoembolization can be safely performed in patients with hepatocellular carcinoma invading the main portal vein and may improve the overall survival. Radiology.

[CR9] Demirjian A, Peng P, Geschwind JF, Cosgrove D, Schutz J, Kamel IR, Pawlik TM (2011). Infiltrating hepatocellular carcinoma: seeing the tree through the forest. J Gastrointest Surg Off J Soc Surg Alimentary Tract.

[CR10] European Association for the Study of the Liver, European Organisation for Research and Treatment of Cancer (2012). EASL-EORTC clinical practice guidelines: management of hepatocellular carcinoma. J Hepatol.

[CR11] Ferlay J (2015). Cancer incidence and mortality worldwide: sources, methods and major patterns in GLOBOCAN 2012. Int J Cancer.

[CR12] Hucke F (2014). The ART-strategy: sequential assessment of the ART score predicts outcome of patients with hepatocellular carcinoma re-treated with TACE. J Hepatol.

[CR13] Ikai I (2005). Report of the 16th follow-up survey of primary liver cancer. Hepatol Res Off J Jpn Soc Hepatol.

[CR14] Kim HY (2012). Severity and timing of progression predict refractoriness to transarterial chemoembolization in hepatocellular carcinoma. J Gastroenterol Hepatol.

[CR15] Kim YI (2014). Long-term outcomes of second treatment after initial transarterial chemoembolization in patients with hepatocellular carcinoma. Liver Int.

[CR16] Kudo M (2011). Management of hepatocellular carcinoma in Japan: consensus-based clinical practice guidelines proposed by the Japan Society of Hepatology (JSH) 2010 updated version. Dig Dis.

[CR17] Kudo M, Arizumi T, Ueshima K (2014). Assessment for retreatment (ART) score for repeated transarterial chemoembolization in patients with hepatocellular carcinoma. Hepatology.

[CR18] Kudo M (2014). JSH consensus-based clinical practice guidelines for the management of hepatocellular carcinoma: 2014 update by the Liver Cancer Study Group of Japan. Liver Cancer.

[CR19] Kwak HW (2014). Clinical outcomes of a cohort series of patients with hepatocellular carcinoma in a hepatitis B virus-endemic area. J Gastroenterol Hepatol.

[CR20] Lee SD, Lee B, Kim SH, Joo J, Kim SK, Kim YK, Park SJ (2016). Proposal of new expanded selection criteria using total tumor size and (18)F-fluorodeoxyglucose—positron emission tomography/computed tomography for living donor liver transplantation in patients with hepatocellular carcinoma: the National Cancer Center Korea criteria. World J Transplant.

[CR21] Llovet JM (2005). Updated treatment approach to hepatocellular carcinoma. J Gastroenterol.

[CR22] Llovet JM (2008). Design and endpoints of clinical trials in hepatocellular carcinoma. J Natl Cancer Inst.

[CR23] Park JW (2015). Global patterns of hepatocellular carcinoma management from diagnosis to death: the BRIDGE Study. Liver Int.

[CR24] Takayasu K (2006). Prospective cohort study of transarterial chemoembolization for unresectable hepatocellular carcinoma in 8510 patients. Gastroenterology.

[CR25] Trevisani F, Cantarini MC, Wands JR, Bernardi M (2008). Recent advances in the natural history of hepatocellular carcinoma. Carcinogenesis.

[CR26] Yamanaka K (2012). Early evaluation of transcatheter arterial chemoembolization-refractory hepatocellular carcinoma. J Gastroenterol.

